# Dual-Stimuli Injectable Platforms for Localized Breast Cancer Therapy

**DOI:** 10.1021/acs.nanolett.6c00777

**Published:** 2026-04-26

**Authors:** Celia Nieto, Álvaro González-Garcinuño, Antonio Tabernero, Eva M. Martín del Valle

**Affiliations:** † Department of Chemical Engineering, 16779 University of Salamanca, Plaza de los Caídos s/n, 37001 Salamanca, Spain; ‡ Institute of Biomedical Research, Hospital Virgen de la Vega, Paseo San Vicente 58-182, 37007 Salamanca, Spain

**Keywords:** breast cancer, drug delivery system, injectable hydrogel, liposomes, thermoresponsive, pH-responsive

## Abstract

Locoregional breast cancer recurrence remains a significant therapeutic challenge, largely driven by limited drug selectivity and toxicity to surrounding healthy tissues. This Mini-Review examines the emerging potential of pH- and temperature-responsive formulations as injectable drug delivery systems designed to achieve preferential targeting of malignant cells. A range of advanced platforms—from smart hydrogels to hybrid constructs such as pH-sensitive liposomes embedded within thermoresponsive hydrogel matrices—are discussed, with emphasis on key design principles and synthesis strategies enabling environmental responsiveness. Among the technologies reviewed, liposome-in-hydrogel hybrid systems—still largely unexplored in breast cancer therapy—stand out for their capacity to enhance encapsulation of lipophilic therapeutics, improve formulation stability, streamline manufacturing, and provide sustained, spatially controlled drug release. Finally, critical physicochemical, mechanical, and biological characterization studies that are needed to rigorously evaluate the translational and clinical potential of these materials for the treatment of locoregional recurrent breast cancer are outlined.

## Graphical abstract

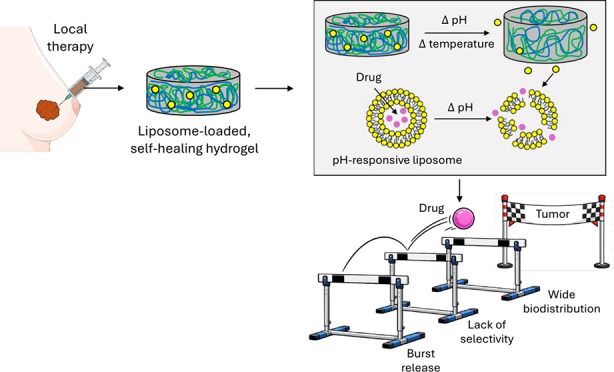

Breast cancer (BC) is the second most frequently diagnosed malignancy worldwide, with approximately 1.4 million new cases reported each year. Despite significant advances in therapeutic strategies, patients diagnosed with BC are commonly treated with lumpectomy followed by systemic adjuvant chemotherapy to reduce the risk of tumor recurrence and distant metastasis.^1,2^ However, the lack of selectivity of most chemotherapeutic agents results in substantial toxicity to healthy tissues, leading to severe adverse effects. Consequently, both academic and pharmaceutical researchers are actively developing novel stimuli-responsive drug delivery systems (DDS) designed to enhance the selective delivery of anticancer drugs and improve their therapeutic efficacy.^3^ Due to the hypoxic and highly metabolic nature of tumor tissues, which promotes lactic acid accumulation and a reduced extracellular pH (6.5–6.8), pH-sensitive DDS have attracted considerable interest as an effective strategy to enhance drug accumulation within the tumor microenvironment (TME), including in drug-resistant cells.^4,5^ Nevertheless, despite the advantages offered by these “smart” DDS—formulated by incorporating pH-sensitive materials—systemic administration remains challenging because of the potential premature release of chemotherapeutics in circulation, suboptimal tumor specificity, and high angiogenic activity in tumors, which contributes to recurrence risk.^6,7^

In this context, localized DDS—including micelles, nanoparticles, and hydrogels—offer a powerful approach to improve the efficacy and safety of cancer therapy by enhancing drug solubility and stability, and maximizing its concentration at the target site.^1,8,9^ Among these systems, hydrogels have garnered particular attention due to their high porosity and tunable structure, which enable controlled and sustained drug release. Injectable hydrogels are especially promising for local BC therapy because they can be administered in a liquid form and subsequently undergo *in situ* gelation at body temperature. This property allows prolonged and localized release of encapsulated chemotherapeutics at the pathological site, thereby enhancing antitumor effectiveness, reducing recurrence or metastasis and, in addition, enabling precise filling of postsurgical cavities.^6,10^

Considering the above, drug delivery composites that integrate a pH response with thermosensitive hydrogels are captivating the scientific community owing to their synergist and complementary properties. The components of these systems should interact in a way that enhances both their structural integrity and functional performance, improving mechanical stability while modulating the swelling–deswelling behavior and rheological properties of the hydrogels, thereby enabling fine-tuning of drug release profiles within the hybrid system.^11^ Figure 1 shows the main advantages of the proposed pH-responsive DDS and highlights the reasons why they may be effective for the local treatment of BC.

**Figure 1:**
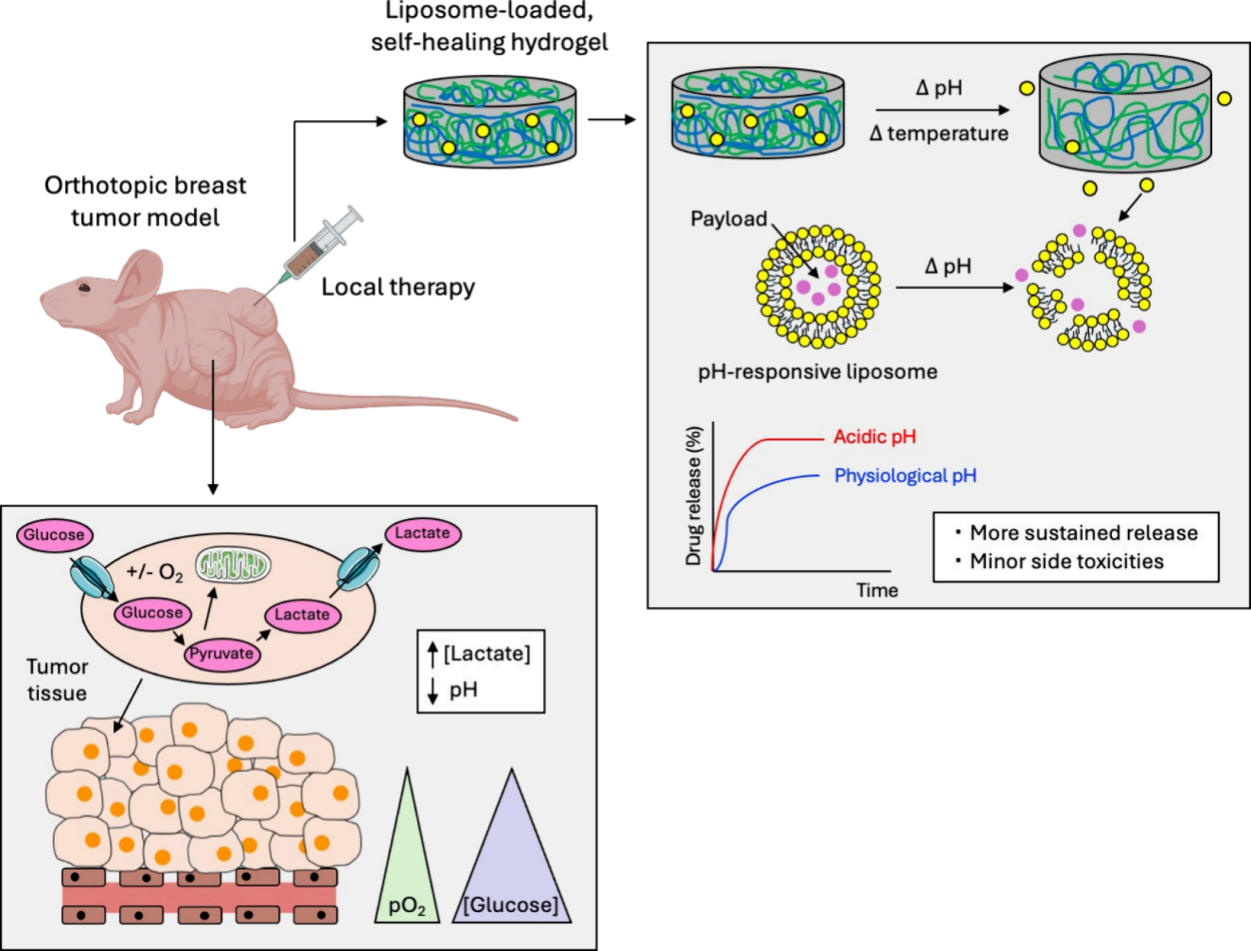
Proposed strategies for local treatment of BC involving injectable systems that exploit lactate accumulation resulting from the Warburg effect in cancer cells, which leads to the acidification of the extracellular microenvironment. These approaches include the development of multi-stimuli-responsive DDS, triggered by pH and temperature, based on hydrogels and liposome-loaded hydrogels.

This Mini-Review highlights the promise of multi-stimuli-responsive DDS for local treatment of BC, with particular emphasis on strategies to engineer hybrid platforms that integrate pH-sensitive liposomes within thermoresponsive hydrogels. While conventional liposomal formulations are frequently constrained by limited stability and rapid degradation in physiological environments, their incorporation into injectable hydrogel matrices offers a compelling solution to overcome premature drug leakage. This hybrid approach not only enhances formulation stability but also enables spatially confined, sustained drug release—positioning these systems as a highly promising avenue for the development of next-generation locoregional BC therapies.

## Thermosensitive Hydrogels and Gelling Mechanism

Thermosensitive hydrogels are a suitable option for filling cavities after resection surgery. Hydrogels can absorb large amounts of water and swell, and they can be synthesized through either physical or chemical cross-linking. Physically cross-linked hydrogels are formed through weak interactions, (e.g., van der Waals forces) that can be triggered by external stimuli. In contrast, chemically cross-linked hydrogels rely on covalent bonds, resulting in stronger interactions. In the case of thermosensitive systems, physically cross-linked hydrogels can be formed in response to temperature changes, enabling reversible gelation and conferring injectable properties that are generally not achievable with other types of cross-linking.^12,13^

Understanding the upper critical solution temperature (UCST) and lower critical solution temperature (LCST) is crucial for describing polymer thermosensitivity. The UCST is the highest temperature that a polymer requires to achieve being fully miscible in a solvent. Under UCST, different phases are formed. Conversely, the LCST is the lowest temperature at which the polymer is fully soluble; above the LCST, phase separation occurs and the polymer becomes insoluble. As a result, polymers exhibiting UCST behavior form hydrogels upon cooling, whereas polymers with LCST behavior undergo gelation upon heating. Obviously, polymers with a LCST near physiological temperature are particularly attractive for the development of injectable hydrogels for biomedical applications.^14,15^

LCST-type materials are typically synthetic amphiphilic copolymers composed of hydrophobic and hydrophilic blocks arranged in various configurations (Figure 2). Generally, in aqueous environments below the LCST, hydrogen bonds form between water molecules and the hydrophilic segments of the polymer, maintaining solubility. As temperature increases, these bonds weaken and hydrophobic interactions become dominant. This leads to water release, polymer chain contraction, and enhanced polymer–polymer interactions, promoting the formation of micelles with hydrophobic cores and hydrated shells. Above the LCST—and often above a critical concentration (critical micellar concentration (CMC))—these micelles aggregate, leading to a sol–gel transition and hydrogel formation. This process can be described as micellization followed by micellar packing, which may involve individual micelles, intermicellar bridging, and eventual micellar collapse.^16,17^ This mechanism is illustrated in Figure 2.

**Figure 2:**
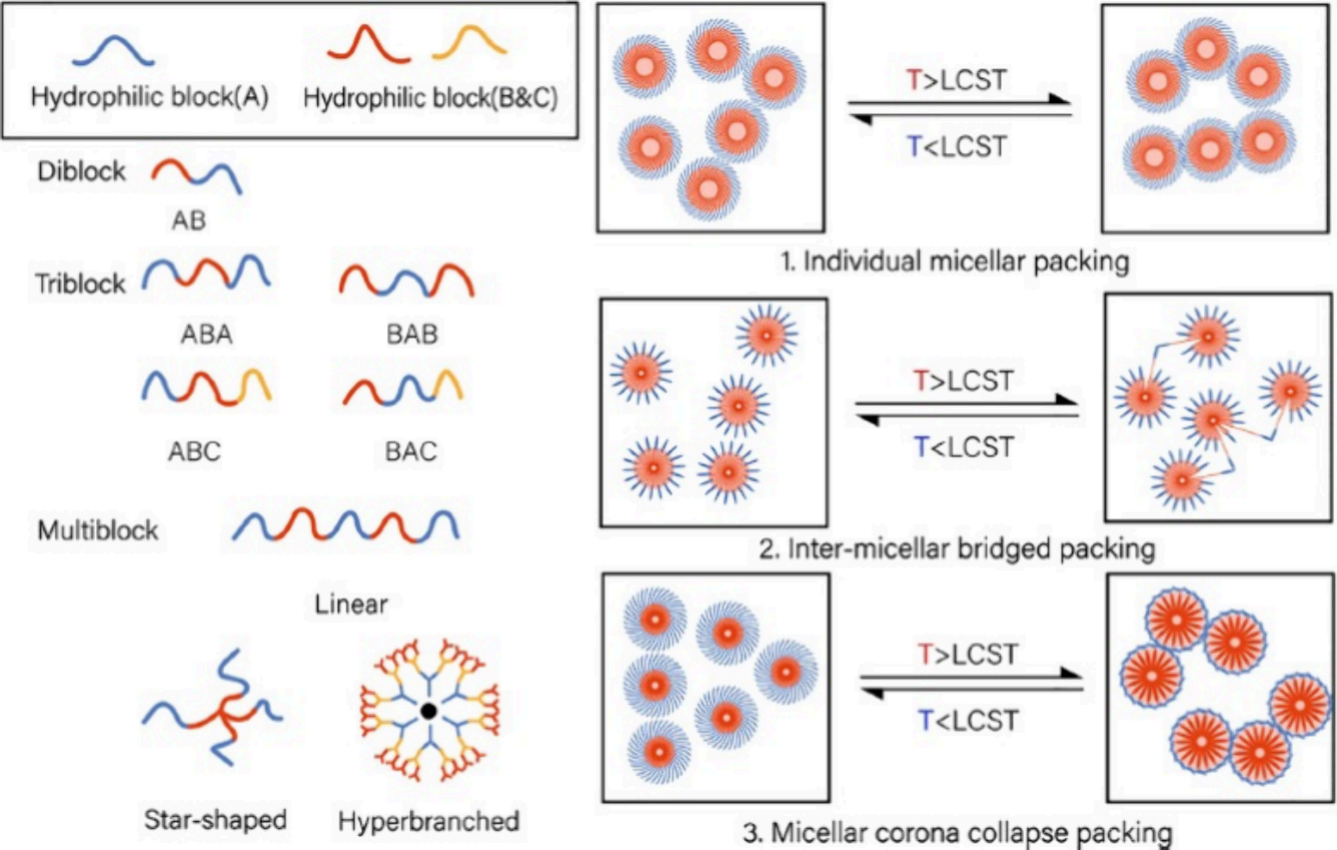
Representative configurations of synthetic amphiphilic copolymers with thermosensitive properties and their mechanisms of micellization. Reproduced from ref 16, under a CC BY-NC-ND 4.0 license.

In this context, Table 1 summarizes several synthetic polymers with LCST values close to the physiological temperature, along with a description of their chemical structures. It is important to note that the LSCT can be tuned by modifying parameters such as molecular weight, polymer concentration, and the ratio of hydrophobic/hydrophilic blocks or by blending with other polymers.

**Table 1:** Various Examples of Thermosensitive Polymers (Synthetic and Natural/Synthetic)^a^

**Polymer(s) (Natural/Synthetic)**	**Structure/Type**	**Information**	**Ref.**
PNIPAm (Synthetic)	Repeating units of *N*-isopropylacrylamide. The amide block is hydrophilic, whereas the isopropyl block is hydrophobic.	Low biocompatibility and biodegradability. It is usually copolymerized or mixed with other polymers, modifying as a result the LCST (LCST usually 32 °C).	^18^

Pluronics (poloxamers) (Synthetic)	Triblock polymers with a central hydrophobic block with POP and two hydrophilic blocks of POE.	Depending on the concentration and the formulation (LCST approximately 15–40 °C).	^19^

Jeffamines (poly(etheramines)) (Synthetic)	Hydrophilic PEO and hydrophobic PPO.	Depending on the concentration and the formulation (LCST approximately15–30 °C).	^20^
Hyaluronic acid/JeffamineM2005 (Natural/Synthetic)	Grafting JeffamineM2005 (PPO/PEO ratio 29/6) to Hyaluronic acid	Performing EDC/NHS (hydrochloride/*N*-hydroxysuccinimide) carbodiimide reaction) with different solvents to control the gelation temperature (LCST 20–60 °C)	^21^

Alginate/PF127 (Natural/Synthetic)	Physical entanglement by mixing the polymers.	Depending on the ratio, gelation was or was not produced. The gelation temperature can be controlled (LCST 17–26 °C).	^22^

Chitosan/β-GP (Natural/Synthetic)	β-GP acts as a cross-linker.	Thermosensitive hydrogel for *in situ* treatment of ulcerative colitis with puerarin as encapsulate drug. The inflammation was reduced (LCST around 37 °C).	^23^

a Abbreviations: **PNIPAm**, Poly­(*N*-isopropylacrylamide); **POP**, polyoxypropylene; **POE**, polyoxyethylene; **PEO**, poly­(ethylene oxide); **PPO**, poly­(propylene oxide); **PF127**, Pluronic F-127; **β-GP**, β-Sodium glycerophosphate.

On the other hand, natural polymers (such as polysaccharides) offer significant advantages for biomedical applications due to their inherent biocompatibility and biodegradability. Nevertheless, they typically form gels upon cooling (e.g., gellan gum or κ-carrageenan) and generally do not exhibit LCST behavior.^24,25^

However, polysaccharides can be engineered to display LCST-type thermosensitivity through several strategies.^26^ One approach involves finding proper cross-linker-polymer combinations; for instance, chitosan and β-sodium glycerophosphate can form a hydrogel with a LCST close to physiological temperature.^23^ More commonly, thermosensitive behavior is introduced by mixing polysaccharides with synthetic LCST-type polymers. This can be achieved by physical blending (which promotes intermolecular interactions between polymer chains),^22^ by forming interpenetrating polymer networks (IPNs) (where one polymer is entangled within a chemically cross-linked network or several polymers are cross-linked),^27^ or by synthesizing graft copolymers (in which synthetic thermoresponsive chains are chemically attached to a polysaccharide backbone).^21^ In grafted systems, the LCST of the resulting copolymer can be tuned by modifying parameters such as branching and degree of substitution. In all cases, the LCST can vary significantly depending on certain factors, including polymer molecular weight, the ratio of the compounds involved, and the degree of substitution. Representative examples of these systems are summarized in Table 1.

## Development of pH-Sensitive Hydrogels and Liposomes

Exploiting pH gradients within the body has emerged as a powerful strategy to achieve spatially controlled drug release. Differences between physiological tissues, tumor microenvironments, and intracellular compartments create opportunities for the design of DDS that remain stable under normal conditions yet activate selectively at diseased sites. In this context, pH-responsive hydrogels and liposomes have gained considerable attention as versatile platforms capable of improving drug selectivity, enhancing local retention, and minimizing systemic toxicity.^28^

### pH-Sensitive Hydrogels: Design Principles and Moieties

pH-responsive hydrogels are generally built from polymers containing weak acidic (polyacids) or weak basic (polybases) groups, whose ionization state depends on the local pH.^29,30^ Ionization generates electrostatic repulsion within the network, increasing osmotic pressure and water uptake, thereby promoting swelling and drug diffusion.^31^

Polyacids include carboxylic acid-bearing monomers such as acrylic acid or methacrylic acid, sulfonamides, anionic polysaccharides (e.g., alginate, pectin), and anionic polypeptides. Polybases, in contrast, comprise polymers containing amine-, pyridine-, or imidazole- groups, as well as cationic polysaccharides such as chitosan.^29,30^

Carboxylated networks such as poly­(acrylic acid) (PAA), poly­(methacrylic acid) (PMAA), and their copolymers with hydrophilic backbones (e.g., cellulose derivatives, acrylamide) are widely used for oral and parenteral delivery. In these systems, swelling is minimized under acidic conditions and enhanced near neutral pH values.^32^ For example, cellulose derivative/pectin–PMAA hydrogels show minimal cytarabine or insulin release at pH 1.2, but pronounced release at pH 6.8–7.4, providing gastric protection and sustained intestinal delivery.^33^ Semi-interpenetrating polymer networks (semi-IPNs) and copolymers such as microcrystalline cellulose–methacrylic acid or poly­(acrylamide-*co*-acrylic acid) also exhibit high water uptake and strong pH-dependent swelling while maintaining favorable rheological and mechanical properties.

Cationic systems based on primary or tertiary amines (e.g., 2-aminoethyl methacrylate, dimethylaminoethyl methacrylate) display complementary behavior, swelling upon protonation under mildly acidic conditions, enabling acidosis sensing or tumor-targeted release.^34^ Incorporation of these ionizable motifs into nanogels or composite systems (e.g., sodium alginate/PEG-*g*-chitosan or PAA cores coated with liposomes) allows precise tuning of swelling kinetics and controlled release profiles under physiologically relevant pH changes.^35^

Synthesis of these smart matrices typically involves free-radical polymerization of ionizable monomers in the presence of cross-linking agents such as glutaraldehyde, formaldehyde, dialdehydes, epoxy compounds, or divinyl compounds (e.g., *N,N*′-methylenebis­(acrylamide) (MBA)). Polymerization can be thermally initiated (e.g., using ammonium persulfate) or photoinitiated via UV irradiation, forming covalent bonds that define the permanent network structure.^36^ To enhance mechanical strength or introduce multifunctionality, IPN synthesis is frequently employed, wherein a second polymer is polymerized within an existing network without necessarily forming covalent bonds between them. Additionally, for natural polymers such as chitosan or alginate, physical cross-linking via ionic gelation or polyelectrolyte complexation is a common alternative, avoiding toxic initiators and preserving biocompatibility.^37^

Across these systems, the type, density, p*K*_a_, and distribution of pendant ionic groups are critical determinants of swelling behavior and switching pH. Table 2 and Figure 3 summarize the most representative ionizable moieties used for each polymer and their corresponding phase transitions.

**Figure 3:**
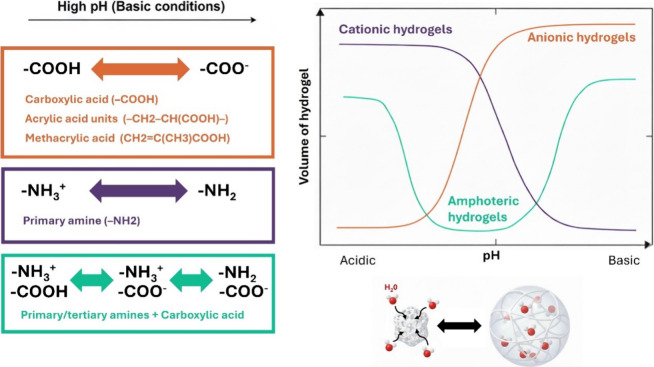
Some representative ionizable moieties and their associated phase transitions. Changes in pH alter the ionization state of the hydrogels, modulating electrostatic repulsion within the network. This can either expand the polymeric chains, promoting swelling, or reduce repulsion, leading to chain compaction. In amphoteric hydrogels, which contain both acid and basic moieties, two distinct phase transitions can occur corresponding to the ionization of each type of functional group. Reproduced from ref 38, under a CC BY 4.0 license.

**Table 2:** Ionizable Moieties Enabling pH-Responsive Hydrogel Behavior^a^

**Polymer**	**pH-sensitive moiety (group)**	**Typical behavior vs pH**	**Ref.**
PAA, P(MAA), Na-CMC, pectin	Carboxylic acid (−COOH)	Deprotonation above p*K*_a_	^32^
		Anionic swelling at pH > 5	

Poly(acrylamide-*co*-acrylic acid)	Acrylic acid units (−CH_2_–CH(COOH)−)	Increased charge density and swelling with rising pH (pH > 6)	^39^

Cellulose/microcrystalline cellulose IPNs	Methacrylic acid (CH_2_=C(CH_3_)COOH)	Collapsed at pH ≈ 1–2; swollen and releasing at pH ≥ 6.8	^33^

Chitosan, PEG-*g*-chitosan	Primary amine (−NH_2_)	Protonation in acidic media → cationic, swollen (pH < 6)	^40^

AEMA-, DMAEMA-modified poly(HEMA)	Primary/tertiary amines	Very sensitive to small changes at physiological pH (pH ≈ 7.4). Ideal for tumor targeting	^34^

Anionic polysaccharides (alginate)	Carboxylate on uronic acids	Ionization at neutral pH enhances degradation or swelling	^35^

a Abbreviations: **PAA**, poly­(acrylic acid); **P­(MAA)**, Polymethacrylic acid; **Na-CMC**, Sodium carboxymethyl celullose; **IPNs**, Interpenetrating polymer networks; **PEG**, Polyethylene glycol; **AEMA**, 2-Aminoethyl methacrylate; **DMAEMA**, 2-(Dimethylamino)­ethyl methacrylate; **HEMA**, 2-Hydroxyethyl methacrylate.

### pH-Sensitive Liposomes

pH-sensitive liposomes are formulated to remain stable at physiological pH (around 7.4) but to destabilize, fuse, or disassemble under the mildly acidic conditions characteristic of tumors and endosomal/lysosomal compartments (pH 4.5–6.8).^41^

A classical approach blends a cone-shaped fusogenic lipid, typically dioleoyl­phosphatidyl­ethanol­amine (DOPE) or other phosphatidyl­ethanol­amines, with an acidic stabilizer such as cholesteryl hemisuccinate (CHEMS) or other carboxylated amphiphiles.^42^ At neutral pH, the ionized acidic components stabilize the bilayer’s lamellar phase. Upon acidification, protonation reduces electrostatic stabilization, allowing DOPE to adopt its intrinsic hexagonal phase, promoting membrane fusion, and cargo release. Stability in serum can be further enhanced by incorporating anionic diolein, Tween-80, or oleyl alcohol.^43^

More advanced designs use pH-labile linkers—such as hydrazone, acetal, or Schiff base—within PEG–lipid or lipid–drug conjugates. For example, PEG2000–hydrazone–stearate on liposome surface provides long circulation at physiological pH but cleaves in acidic environments, exposing cell-penetrating peptides and enhancing tumor uptake and cytosolic delivery.^44^ pH-sensitive prodrugs embedded in the bilayer enable selective release of highly cytotoxic compounds in the acidic tumor microenvironment while remaining stable in blood.^45^

An alternative strategy involves coating liposomes with “smart” polymers, such as polymethacrylic acid copolymers, which undergo pH-dependent conformational changes or swelling that triggers layer detachment and cargo release. Coatings are typically applied via electrostatic deposition, where preformed liposomes (often possessing a net negative or positive surface charge) are added to a dilute solution of the pH-responsive polymer under controlled stirring, allowing polymer chains to attach through electrostatic, hydrogen bonding, or hydrophobic interactions. Layer-by-layer (LbL) assembly is another approach, where alternating layers of oppositely charged polyelectrolytes are deposited to form a nanoshell.^46^

Overall, key design variables for pH-sensitive liposomes include (i) the proportion of fusogenic helper lipids (DOPE), (ii) the choice of acidic stabilizers or pH-labile linkers with appropriate p*K*_a_, (iii) PEGylation density, and (iv) the incorporation of targeting ligands to enhance tumor accumulation and endosomal escape.^41^

Table 3 summarizes the main pH-sensitive liposomal formulations, highlighting their main components and mechanistic response to acidic environments.

**Table 3:** pH-Sensitive Liposomal Formulations^a^

**System**	**Main pH-sensitive components**	**Mechanistic feature at low pH**	**Ref.**
Classical PE-based pH-sensitive liposomes	DOPE + CHEMS (or other PE + carboxylic stabilizer)	Protonation of acidic lipid → DOPE hexagonal phase, fusion and release	^42^

Anionic pH-sensitive liposomes	Diolein/CHEMS	Stable at pH 7.4; rapid aggregation and calcein release at pH ≈ 5	^47^

Novel CHEMS/Tween-80-based systems	PC/PE or PC + CHEMS + Tween-80 ± OAlc	Improved pH sensitivity and serum stability vs DOPE formulations	^47^

PEG–hydrazone–stearate CPP-modified liposomes (CPPL)	PEG2000–Hz–stearate + CPP–stearate + conventional phospholipids	Acid-labile PEG detachment and enhanced CPP-mediated tumor penetration	^44^

Folate-coated long-circulating pH-sensitive liposomes	PEGylated phospholipids + pH-sensitive lipid mix + folate-PEG	Long circulation and enhanced tumor uptake in folate-positive tumors	^48^

Multiliposomal complexes with ampholytic cholan-24-oic derivative	Anionic liposomes with ampholytic cholan derivative + PEGylated cationic carrier liposomes	Rapid cargo release at tumor-relevant acidic pH, low cytotoxicity	^49^

a Abbreviations: **PE**, Phosphatidylethanolamine; **DOPE**, 1,2-dioleoyl-*sn*-glycero-3-phospho­ethanol­amine; **CHEMS**, Cholesteryl hemisuccinate; **PC**, Phosphatidyl­choline; **CPP**, Cell-penetrating peptide.

## Temperature and pH-Sensitive Systems

Combining temperature and pH responsiveness within a single delivery platform represents a powerful approach to achieve spatiotemporal control over drug release. By integrating multiple physiological triggers, these systems can remain injectable and minimally invasive during administration while undergoing *in situ* gelation and stimulus-activated drug release once exposed to the tumor microenvironment. Such dual-responsive materials are particularly attractive for locoregional therapies, where precise control over retention, release kinetics, and environmental activation is essential.

### pH- and Temperature-Sensitive Hydrogels

A widely adopted strategy to impart dual pH- and temperature-sensitivity to polymeric carriers involves grafting thermoresponsive polymers with pH-responsive functional moieties. For example, carboxyl groups can be grafted onto PF127 using an oligosaccharide spacer to introduce pH sensitivity, although this modification can alter the polymer’s LCST.^50^ A similar approach was followed by Rungrod et al. (2025),^51^ who grafted PF127 with N-succinyl chitosan, and by García-Sobrino et al. (2024),^52^ who incorporated 2-(diisopropylamino)­ethyl methacrylate (DPAEMA) into a temperature-sensitive polymer (N-vinyl caprolactam). Another strategy, proposed by Ma et al. (2023),^53^ combined RAFT polymerization with host–guest interactions using cyclodextrins.

While various multistimuli systems have been engineered for BC therapy—integrating, for instance, photothermal, magnetic, and pH-responsive functionalities^54^—the use of pH/temperature-sensitive hydrogels for localized treatment remains relatively unexplored, despite their significant therapeutic potential. Recently, Luo and Hu^55^ developed a dual pH- and temperature-sensitive hydrogel based on *N*-isopropyl­acrylamide, itaconic acid, and chitosan (cross-linked with β-GP) to improve biocompatibility and tune the LCST. The chemotherapeutic agent 5-fluorouracil (5FU) was entrapped within the hydrogel. Swelling studies indicated a clear response to pH and temperature variations. Drug release was faster at 40 °C and pH 5.8 (40% in 144 h) compared to 40 °C and pH 7.4 (20% in 144 h). Moreover, the hydrogel exhibited good biocompatibility in hemolysis tests. *In vitro* studies showed that the unloaded hydrogel was nontoxic toward BT-20 and HCC1937 cells, whereas both free 5FU and 5FU-loaded hydrogel (at a drug concentration of 4 μg·mL^–1^) reduced cell viability to approximately 15% after 24 h. *In vivo* assays conducted in three groups (free hydrogel, free drug, and drug-loaded hydrogel; *n* = 6 per group) revealed a significant reduction in tumor volume for both the free drug and the loaded hydrogel (33% reduction compared to the free hydrogel). Importantly, no pathological alterations were observed in heart, liver, spleen, lungs, or kidney tissues 28 days after administration of the loaded hydrogel (*p* < 0.05 vs free drug and free hydrogel). Additionally, no significant changes in body weight were observed in animals treated with either the free or loaded hydrogel. In contrast, mice treated with the free drug exhibited weight loss, as well as signs of toxicity such as erythema and diarrhea.

Table 4 and Figure 4 show some results concerning the use of pH- and temperature-sensitive hydrogels.

**Figure 4:**
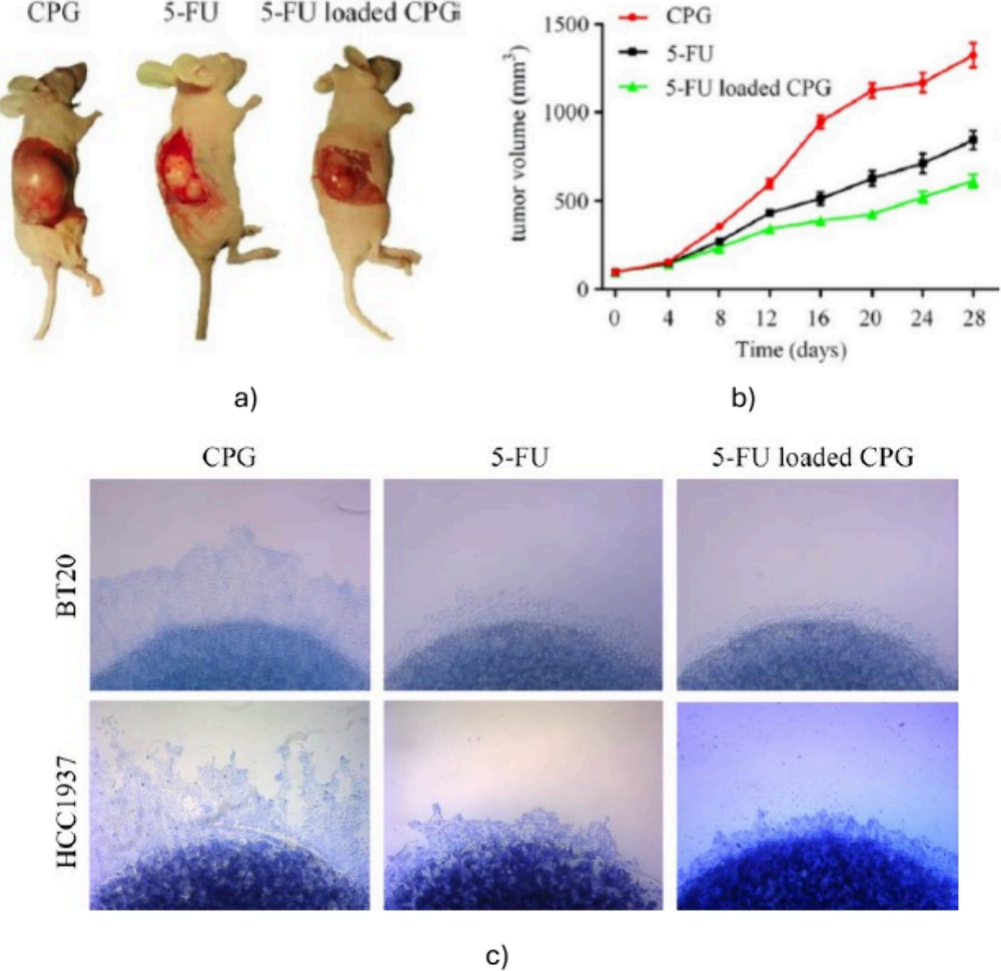
Results of a pH/temperature-sensitive hydrogel loaded with 5FU for BC treatment. (a) Picture of mice after 28 days of treatment with hydrogel alone (CPG, left), free 5FU (middle), and 5FU-loaded hydrogel. Tumor volume is visibly reduced in mice treated with the 5FU-loaded hydrogel. (b) Notably, mice receiving free 5FU exhibited adverse effects, such as irritation, swelling, and diarrhea. (c) Results of collagen sprout growth assay assessing cell migration and proliferation in BT-20 and HCC1937 BC cells. Sprout formation was markedly reduced with the 5FU-loaded hydrogel, indicating effective inhibition of cancer cell migration and proliferation. (a–c) Reproduced from ref 55, under a CC BY-NC-ND 4.0 license.

**Table 4:** Multi-stimuli (pH- and Temperature-Sensitive) Drug Release Systems^a^

**Type (sensitivity)**	**Components**	**Information**	**Ref.**
Hydrogel (pH, T)	*N*-Isopropylacrylamide with itaconic acid and chitosan (cross-linked with β-GP).	BC therapy. The addition of itaconic acid and chitosan with β-GP improved biocompatibility and helped in reaching a correct LCST. Adequate results *in vivo* and *in vitro* concerning biocompatibility, drug release, and antiproliferation.	^55^

Hydrogel (pH, T)	*N*-Succinyl chitosan (pH responsive) and PF127 (T responsive).	Sprayable system for wound healing with an entrapped antioxidant. Gelation at 35 °C. Hydrogel stable after 120 days of storage at pHs lower than 5. 50–80% of the drug was released in less than 24 h.	^51^

Hydrogel/liposomes (T)	Paclitaxel encapsulated in liposomes (no pH-sensitive) with paclitaxel. Liposomes entrapped in two poloxamers (PF127 and P188) to obtain a T-response.	Injectable hydrogel for pancreatic cancer. The tumor was suppressed in 12 h. The release was significantly prolonged. Low toxicity toward lungs and heart; no necrosis and no inflammation.	^58^

Hydrogel/liposomes (pH, T)	Arctiginin encapsulated in liposomes pH-sensitive based on hydrazone. Liposomes entrapped in two poloxamers (PF127 and P188).	The drug was only released at the targeted pH (pH 5.0). The cytotoxicity was improved.	^62^

a Abbreviations: **PF127**, Pluronic F-127; **β-GP**, β-Sodium glycerophosphate; **P188**, Poloxamer 188.

Nevertheless, as noted previously, these methodologies usually involve lengthy, multistep polymerization processes to functionalize the polymer, which complicates the simultaneous control of pH sensitivity and LCST. Additionally, drug release kinetics are difficult to regulate, as they largely depend on polymer degradation in response to pH. This process can itself alter local pH and may potentially induce inflammation in physiological environments.

### pH- and Temperature-Sensitive Hydrogels and Liposomes

Given the limitations of grafting-based multistimuli systems, an alternative—although less widely explored—is the entrapment of pH-sensitive carriers, such as liposomes, within thermosensitive polymers.

Entrapping conventional (nonsensitive) liposomes within polymeric matrices has been widely investigated, as it offers several advantages: the additional polymeric barrier can retard drug release and prevent leakage, enhance biocompatibility, and confer versatility by allowing the encapsulation of both hydrophilic and hydrophobic drugs.^56,57^ Some of these systems have even progressed to phase II clinical trials, including a topical liposomal amphotericin B gel used for the treatment of cutaneous leishmaniasis and a topical liposomal gel formulation of a vitamin B12 analogue (HL-009) for the management of atopic dermatitis.

The most common way to synthesize such composite systems is straightforward: liposomes are directly incorporated into the hydrogel network (Figure 5), promoting interactions between the two components. This strategy enables a sequential synthesis, avoiding long grafting or cross-linking procedures. Importantly, interactions between liposomes and the hydrogel should be characterized—mainly via rheology—to determine whether liposome incorporation strengthens or weakens the gel, and how it affects material degradation.

**Figure 5:**
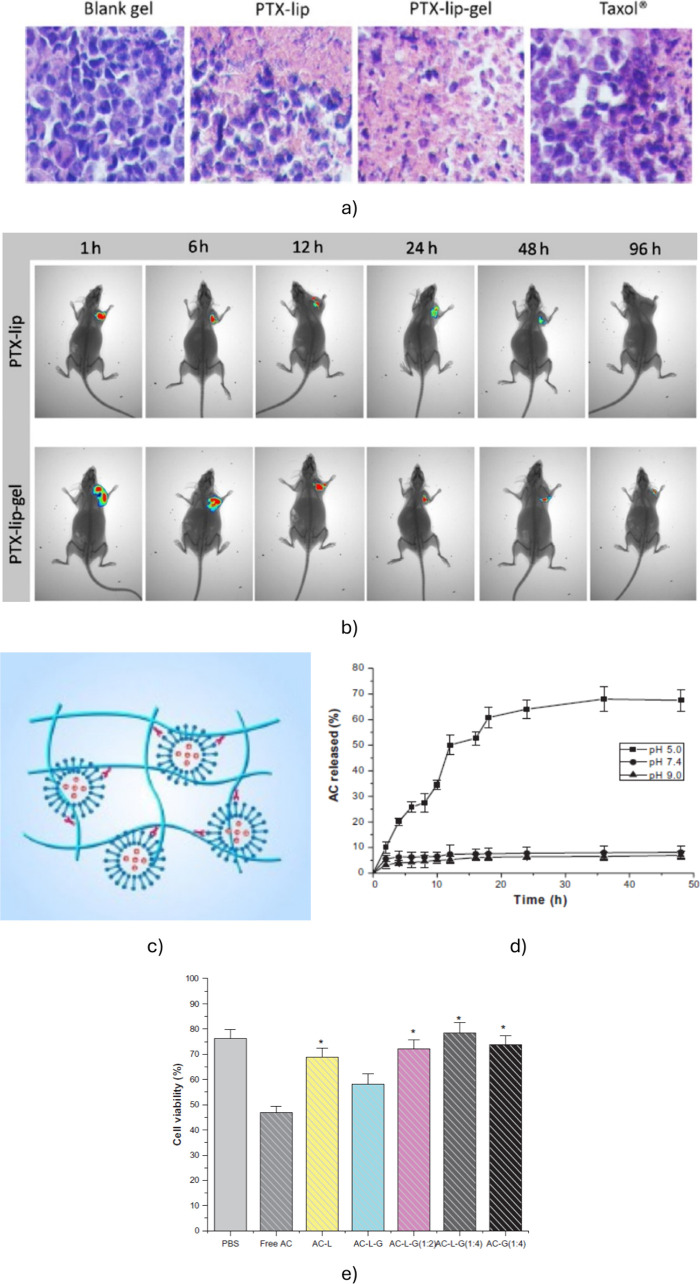
(a) *Ex vivo* histological analysis of tumor sections after 3 days of treatment with hydrogel alone, paclitaxel-loaded liposomes (PTX-Lip), PTX-Lip in hydrogel, and commercial PTX (Taxol). The interstitial space and cytoplasm are more pronounced in PTX-Lip-hydrogel-treated tumors, indicating effective cytotoxicity against S180 ascitic tumor cells. Reproduced with permission from ref 58. Copyright 2016 Elsevier. (b) *In vivo* NIR imaging of mice with tumors, showing the retention of PTX for longer periods when delivered via PTX-Lip in thermosensitive hydrogels compared to PTX-Lip alone. Reproduced with permission from ref 58. Copyright 2016 Elsevier. (c) Schematic representation of the mechanism by which liposomes are entrapped in hydrogels through intermolecular interactions. Reproduced from ref 57, under a CC BY 4.0 license. (d) Controlled release of arctigenin achieved by encapsulating it in liposomes that are further entrapped in a thermosensitive hydrogel. Reproduced from ref 62, under a CC BY-NC license. (e) Improved biocompatibility of arctigenin when delivered via liposomes entrapped in thermosensitive hydrogels compared to free arctigenin. The figure also illustrates the effect of varying the ratios of arctigenin (AC), liposomes (L), and hydrogel (G) on drug delivery performance. Reproduced from ref 62, under a CC BY-NC license.

For injectable hydrogel–liposomes systems, thermosensitivity of the hydrogel is critical. Again, the additional polymeric barrier can delay drug release, but this effect occurs only above the sol–gel transition temperature. Systems with transition temperatures above room temperature maintain injectability. For example, Mao et al.^58^ encapsulated paclitaxel in non-pH-sensitive liposomes and subsequently entrapped them into poloxamers PF127 y P188 to confer temperature responsiveness to the system for pancreatic cancer therapy. Liposome entrapment significantly prolonged drug release (from 20% at 12 h for free liposomes to 80% over the same period) and suppressed tumor growth within 12 days. Specifically, *in vivo* studies conducted in 4 groups (free gel, paclitaxel-loaded liposomes (PTX-lip), paclitaxel-loaded liposomes incorporated into gel (PLG) and free Taxol; *n* = 6 per group) showed that the PLG system degraded within 5 days, while significantly reducing relative tumor volume (0.49) compared to free gel (2.31; *p* < 0.05 vs control). Body weight loss was observed only in mice treated with free Taxol. Histological analyses, based on drug distribution in major organs (heart, liver, spleen, lung, and kidney), demonstrated good *in vivo* biocompatibility and minimal toxicity (Figure 5). For the PLG system, drug concentration in heart, lung, spleen, and kidney remained below the limit of quantification (<50 ng·mL^–1^) after 48 h, whereas a concentration of approximately 600 ng·mL^–1^ was detected in the liver. In contrast, concentrations below the limit of quantification were consistently observed for the PTX-lip formulation. These results suggested that the PLG system provided high local drug concentrations while minimizing systemic side effects.

In the same way, in the context of BC therapy, Li et al.^59^ encapsulated curcumin in liposomes coated with thiolated chitosan. This system formed a gel at 37 °C, exhibited good cytocompatibility, and *in vivo* studies showed reduced recurrence following surgical resection, along with enhanced tissue repair. Concretely, five experimental groups (PBS, free drug, curcumin-loaded liposomes (LC), free hydrogel, and hydrogel containing LC (HLC); *n* = 6 per group) were evaluated. No significant body weight loss was observed in any group, while the longest survival period (>24 days) was recorded for the LC and HLC formulations. As previously noted, this study provided a critical evaluation of *in situ* tumor recurrence. The results demonstrated recurrence rates of approximately 75% for PBS and free hydrogel, 40% for the free drug and LC, and no recurrence for the HLC formulation. Furthermore, no secondary metastases were observed in the HLC-treated group. Histological analysis indicated that most formulations did not induce noticeable side effects; however, vacuolar degeneration and renal necrosis were observed in the group treated with the free drug.

Drug release can be further controlled by combining thermosensitive liposomes with thermosensitive hydrogels. Kong et al.^60^ applied this strategy to pancreatic cancer by incorporating gemcitabine and a photothermal agent. The presence of multiple barriers ensured that drug release occurred only upon laser irradiation. Particularly, *in vitro* results showed that the liposome–gel formulation combined with irradiation exhibited the highest cytotoxicity toward the PANC-1 cell line, achieving approximately 80% antiproliferative effect, compared to around 40% for liposomes and free drug under laser irradiation. In addition, *in vivo* studies conducted in 5 groups (PBS, liposomes (LI), liposomes with laser (LIL), liposome–gel (LIG), and liposome–gel with laser (LIGL); *n* = 4 per group) demonstrated that the LIGL system significantly suppressed tumor growth by approximately 91% (*p* < 0.01) without adversely affecting animal health, as no significant differences in body weight after were observed after 14 days. Histological analysis further revealed that only the LIGL formulation induced apoptosis and necrosis in tumor tissues.

Similarly, paclitaxel-loaded thermosensitive liposomes have been explored for the treatment of ovarian cancer, enabling localized drug accumulation in the peritoneal cavity while allowing precise control over drug release.^61^ *In vivo* evaluation was performed using 5 groups (control, free PTX, liposomal PTX (LPTX), hydrogel with PTX (PTG), and liposome-in-gel (PLTG)). Body weight loss was observed only in the control and free PTX groups after 11 days (with significant differences of *p* < 0.01, *p* < 0.001, and *p* < 0.05 compared to control, PTX, and LPTX, respectively). Moreover, the antitumor effect was assessed by quantifying the number of cells in the peritoneal cavity after 13 days. This analysis showed that the PLTG formulation reduced cell viability by approximately 65% compared with to free PTX (*p* < 0.001) and by 50% compared to LPTX (*p* < 0.05).

Previous works have demonstrated examples of pH- and temperature-sensitive systems for BC therapy, including hydrogels modified for dual responsiveness and thermosensitive hydrogels with entrapped liposomes (Table 2). However, the entrapment of pH-sensitive liposomes within thermosensitive hydrogels for BC treatment remains underexplored.

For instance, Chen et al.^62^ designed hydrazone-based pH-sensitive liposomes encapsulating arctigenin for vaginal administration and incorporated them into poloxamers P188 and PF127 to confer thermosensitivity. Liposome incorporation did not significantly alter the gelation temperature (37–40 °C). Drug release was highly pH-dependent, with 60% released after 20 h at pH 5.0 compared to only 5% at other pH values, and an entrapment efficiency of 94%. *In vitro* cytotoxicity assays using HEK293 cells demonstrated reduced drug toxicity and enhanced release stability (Figure 5). This study highlights the potential of such dual-responsive systems for local therapies, such as BC treatment, by exploiting pathological pH shifts to achieve controlled drug delivery.

Previous works highlight the potential of developing multistimuli (temperature- and pH-responsive) DDS for BC therapy, either through polymer functionalization to create pH- and thermosensitive hydrogels or by entrapping pH-sensitive liposomes within thermosensitive hydrogels. Local injection of these systems ensures drug accumulation within the target cavity, which can be fully filled due to the sol–gel transition at physiological temperature, while drug release occurs selectively in the acidic environment of tumor cells. Furthermore, the incorporation of drug-loaded liposomes enhances structural stability and prolongs drug release.

Besides, the sequential synthesis of the compartments—liposomes and hydrogels—also facilitates the incorporation of multiple therapeutic compounds into different parts of the system. This allows for the design of multi-stimuli-responsive platforms, where additional triggers (such as near-infrared radiation) can be used to control drug release in a straightforward and tunable manner.

### Comparison between Both pH- and Temperature-Sensitive DDS and Their Potential for BC Therapy

As mentioned previously, the conventional treatment for BC involves surgery followed by radiotherapy and/or adjuvant systemic therapies to eradicate residual malignant cells. Nevertheless, adjuvant treatments are often linked to adverse reactions and systemic toxicity. To address these limitations, a variety of DDS have been created to enhance drug targeting efficiency and reduce off target toxicity.^63^ Among these, pH-responsive liposomes have attracted considerable attention as nanocarriers due to their relatively simple preparation, biocompatibility, and low immunogenicity. However, ordinary liposomes exhibit notable drawbacks, including limited stability and premature drug release.^59^

To overcome these issues, the incorporation of drug-loaded liposomes into thermosensitive hydrogels designed for local administration has emerged as a promising strategy. This approach aims to mitigate the limitations associated with both systemic liposome delivery and the rapid, localized release of free drugs from traditional hydrogels. While thermosensitive hydrogels offer advantages such as improved stability, injectability, and stimuli responsiveness, they generally show poor capacity for encapsulating lipophilic drugs and often possess relatively weak mechanical properties.

Therefore, combining pH-responsive liposomes with thermosensitive hydrogels represents a synergistic strategy to address the shortcomings of each system individually. Embedding liposomes within a hydrogel matrix enhances their stability and enables the design of multi-stimuli-responsive DDS, such as those sensitive to both pH and temperature, through selective functionalization of either component. Moreover, the hydrogel network introduces additional mass transfer barriers that contribute to sustained drug release while reinforcing the structural integrity of the formulation. Liposome entrapment may also improve cellular drug internalization. Importantly, these composite systems can typically be synthesized through relatively simple methods based on weak interactions between liposomes and polymer chains, avoiding the need for complex grafting or chemical cross-linking procedures.

## Translational Challenges: Pharmacokinetics, Reproducibility, Sterilization and Scale-Up

Despite the advantages offered by dual-stimuli injectable platforms over conventional systemic therapies, their successful transition into clinical applications requires addressing several critical challenges, including reproducibility, sterilization, and scalable manufacturing. This section provides an overview of these key limitations and potential strategies to overcome them.

### Pharmacokinetic Validation: Local vs Systemic Drug Exposure

One of the main advantages of injectable hydrogel–liposome systems is their ability to maximize drug concentration at the tumor site while minimizing systemic exposure. To validate this, drug distribution should be quantified not only at the target tissue but also in the plasma. Accordingly, *in vivo* studies should determine drug concentration in (i) tumor tissues (to ensure levels remains within the therapeutic window), (ii) peritumoral parenchyma (to assess local diffusion and protection of surrounding healthy tissues), and (iii) plasma (to confirm that the peak of plasma concentration remains below toxicity thresholds). Comparative analysis of plasma concentration–time profiles between systemic and local administration can demonstrate reduced off-target toxicity and improved therapeutic action. This effect can be further supported by calculating the target-to-plasma AUC (area under the curve) ratio, providing evidence of a localized reservoir effect.^64^

Furthermore, PK/PD modeling can be applied to these systems. Compartmental models may be developed to simulate both the hydrogel matrix and surrounding tissues, incorporating diffusion processes and stimuli-triggered release mechanisms (e.g., pH-responsive bursts). Model validation can be achieved by fitting experimental concentration profiles and tumor growth inhibition data (e.g., using a Gompertz approach).^65^ Such approaches enable estimation of optimal dosing intervals and help prevent subtherapeutic exposure that could promote drug resistance.

In addition, PK/PD modeling can inform dosing schedules. While dosing intervals in systemic therapies are often limited by recovery of healthy tissues, in localized systems they are governed by hydrogel degradation and depletion of the liposomal reservoir. Modeling these kinetics may support extending dosing intervals from days to weeks, thereby improving patient compliance.^66^

### Toxicity and Immune Activation

The rational design of hydrogels for successful clinical translation must consider complex and dynamic cell–material interactions, which can influence outcomes such as necrosis, apoptosis, and the activation of undesirable immune responses that may result in local inflammation, macrophage activation, or fibrotic encapsulation.

In this context, hydrogel degradation plays a central role in modulating these effects.^67^ Hydrogels may degrade via enzymatic, hydrolytic, or photolytic pathways, and the degradation rate critically influences immune responses. Thus, hydrogel degradation profile must be carefully adjusted to align with the timing of immune modulation, since if a material persists in the body for a prolonged period, it can trigger continuous immune surveillance and fibrotic encapsulation. By contrast, excessively rapid degradation may release immunostimulatory byproducts.^68^

Therefore, faster degradation may be advantageous for transient applications (e.g., vaccines), whereas slower degradation is generally preferred for chronic disease conditions requiring sustained delivery.

Natural polymers, due to their biodegradable character, typically degrade enzymatically (e.g., alginates via lyases, collagen via collagenases). However, degradation can generate bioactive fragments capable of activating immune receptors, such as Toll-like receptors (TLRs), thereby promoting inflammatory cascades. These effects may depend on parameters such as polymer molecular weight. For example, hyaluronic acid fragments derived from high molecular weight higher polymers (>500 kDa) can activate TLR2 and TLR4, inducing cytokine production.^69^ Similarly, alginate may promote macrophage activation depending on its purity and endotoxin content.^70^

In contrast, synthetic polymers are often considered relatively inert due to the reduced protein adsorption and limited immune recognition (e.g., PEG), which can decrease macrophage adhesion. Nevertheless, their limited biodegradability may result in long-term persistence, potentially affecting tissue remodelling and inducing antipolymer immune responses that alter pharmacokinetics.^71^ Additionally, degradation products of polymers such as or PVA or PEG may contribute to osmotic stress,^68^ while thermosensitive polymers (e.g., PNIPAm, Pluronics) can generate byproducts with potential long-term effects. Importantly, hydrogel properties—including architecture, pore size, and viscoelasticity—can be tuned to modulate cell–material interactions and improve biocompatibility.^67^

### Reproducibility Challenges

The complexity of dual-stimuli-responsive injectable systems introduces challenges in batch-to-batch reproducibility, which is critical for clinical translation. Therefore, strict quality control criteria regarding deviations among batches must be established.

Key parameters that should be consistently monitored for each batch during validation steps include particle size (±10% from the nominal value), zeta-potential (±5 mV), polydispersity index (always below 0.2), encapsulation efficiency (±5% from the nominal value), LCST (±2 °C), and injectability (±10%), in line with commonly accepted guidelines and International Council for Harmonisation of Technical Requirements for Pharmaceuticals for Human Use (ICH) recommendations.^72,73^

### Sterilization Methods

Sterilization of dual-stimuli systems represents a major challenge for clinical translation, as methods must achieve a sterility assurance level (SAL) of 10^–6^ without compromising formulation integrity.^74^

Thermal sterilization (autoclaving) is widely accessible but generally unsuitable for these systems. High temperatures (121 °C) and overpressure promote irreversible aggregation and, in addition, thermal stress induces liposome membrane fluidization and subsequent massive leakage of the encapsulated drugs.^75^ Membrane filtration (0.22 μm) is effective but limited to systems with sufficiently small particle sizes (<180 nm) and does not remove viruses or mycoplasma, requiring strict aseptic conditions that increase manufacturing costs.^76^ Gamma irradiation is suitable for terminal sterilization but may induce polymer degradation through free radical formation, depending on dose.^77^ Alternative approaches, such as ethylene oxide or supercritical CO_2_, are being explored to reduce processing stress; however, their large-scale application remains limited.^78^

### Scale-Up Challenges

Scaling up from laboratory synthesis (milligram scale) to industrial production (kilogram scale) is not straightforward. In dual-responsive systems, physical interactions between liposomes and hydrogels are highly sensitive to processing conditions, making scale-up a critical bottleneck.

Heat transfer is a key factor: while easily controlled at laboratory scale, large-scale reactors may exhibit non-uniform temperature distribution, potentially affecting LCST and rheological properties. Therefore, scale-up strategies should consider dimensionless parameters, such as the Nusselt number, to maintain thermal consistency.^79^

Regarding liposome production, traditional thin-film hydration methods are difficult to scale. Microfluidic mixing has emerged as a promising alternative, enabling better control over particle size distribution and improved reproducibility.^80^

## Challenges and Future Perspectives

This Mini-Review aims to provide a critical overview of the emerging role of pH- and temperature-responsive, injectable DDS—including hydrogels and hybrid liposome–hydrogel platforms—for post-resection BC treatment, discussing briefly the materials commonly employed to synthesize them, their preparation strategies, and their main advantages and limitations.

Locoregional BC recurrence remains a major unmet clinical challenge, primarily because systemic therapies are unable to achieve sufficiently high drug concentrations at the surgical site without inducing dose-limiting systemic toxicity. In this context, injectable stimuli-responsive DDS represent a promising strategy to address this limitation by providing precise drug release and selective targeting of malignant cells while filling the resection cavity.

Individually, pH- and temperature-responsive injectable hydrogels must be carefully designed in terms of hydrophilicity and molecular weight to ensure structural stability and prevent premature drug release caused by polymer degradation or insufficient drug entrapment. Polymer degradation, along with the nature of its metabolic byproducts and the resulting physiological response, must be thoroughly evaluated—particularly for synthetic polymers whose degradation products may alter local pH at the injection site and potentially induce inflammation or tissue damage. These concerns can be mitigated by using natural polymers (e.g., polysaccharides), but these materials often lack a LCST, so more complex modification strategies that frequently involve organic solvents are necessitated. Thus, grafting reactions or polymer blending may be used to tailor LCST values, and comprehensive rheological characterization is mandatory beforehand.

Otherwise, several of these limitations may be addressed by entrapping liposomes within hydrogel matrices. This hybrid approach enhances both hydrogel network stability and liposomal membrane integrity, thereby prolonging drug release through the presence of multiple diffusion barriers. Moreover, the sequential preparation of liposomes and hydrogels broadens the design space for multi-stimuli-responsive drug release systems. However, rheological studies remain crucial to assess liposome–hydrogel interactions, as these may alter injectability and gelation behavior. Such analyses also contribute to identify how the systems are built from a theoretical point of view and should be systematically performed by comparing hybrid formulations with their single-component counterparts. Another key aspect requiring detailed investigation is the drug release pathway. In multi-responsive systems, it is essential to determine whether the drug is first released from liposomes within the hydrogel matrix and subsequently diffuses through the hydrogel, or whether intact liposomes are released prior to drug liberation. For instance, this could be studied using double fluorescent labeling: fluorescein (green) could be chemically attached to the hydrogel polymer chains, while a rhodamine–lipid conjugate (red) could be incorporated into the liposome membranes. Release assays under appropriate conditions, monitored by spectrofluorimetry, would allow measurement of the fluorescence intensity of both compounds over time. This approach could also help to elucidate the influence of polymer degradation on release kinetics, which remains insufficiently understood.

Finally, for both classes of DDS—injectable hydrogels and hybrid liposome–hydrogel platforms—a comprehensive evaluation of the physiological response is indispensable to ensure therapeutic efficacy and continuing safety. To facilitate clinical translation, further studies are required, including *in vivo* locoregional recurrence assessments (in orthotopic murine models in which tumors are removed, while preserving the covering and surrounding skin) and long-term toxicity evaluations; intravital microscopy to better understand real-time system–tissue interactions; and physics-based, multiscale pharmacokinetic compartmental modeling to stablish optimal therapeutic dosing. In addition, both *in vitro* and *in vivo* assays should be conducted to evaluate cytokine production and the activation of macrophages and dendritic cells to analyze their potential effects on immune responses.

Notably, the incorporation of pH-sensitive liposomes within thermosensitive hydrogels for BC therapy remains largely unexplored and represents a highly promising research avenue. This strategy may offer important advantages for precision therapy, including simplified formulation pathways and improved control over spatiotemporal drug release. Importantly, the incorporation of pH-sensitive liposomes within thermosensitive hydrogels for BC therapy remains largely unexplored and represents a highly promising research avenue. By simplifying formulation strategies, improving robustness, and enabling precise spatiotemporal control of drug release, these hybrid injectable depots may significantly advance post-surgical BC therapy.
